# Biomechanical comparison of the use of different surgical suture techniques for continuous loop tendon grafts preparation

**DOI:** 10.1038/s41598-019-57332-8

**Published:** 2020-01-17

**Authors:** Chen Zhang, Tobias Götschi, Xiang Li, Jess G. Snedeker, Sandro F. Fucentese

**Affiliations:** 1 Department of Orthopedics, Balgrist University Hospital, University of Zurich, 8008 Zurich, Switzerland; 2 Department of Bone and Joint Surgery, The Second Affiliated Hospital of Xi’an Jiaotong University, 710004 Xi’an, China; 3 Institute for Biomechanics, Swiss Federal Institute of Technology (ETH) Zurich, 8008 Zurich, Switzerland; 4 ZuriMED Technologies AG, 8008 Zurich, Switzerland

**Keywords:** Tendons, Trauma, Orthopaedics, Preclinical research

## Abstract

We introduce a new approach for a continuous loop tendon-graft preparation, benchmarking it against established graft preparation techniques widely used in conjunction with non-adjustable interference screw fixation. A four-strand bovine tendon graft was prepared using the following graft preparation techniques: standard graft using the baseball stitch technique (M-tech group); continuous loop graft using the GraftLink^TM^ technique (Arthrex-tech group); continuous loop graft using the Kessler anastomosis technique (Kessler-tech group); and continuous loop graft using a Double-Z anastomosis technique (Double Z-tech group). Each group of eight specimens underwent cyclic loading followed by a load-to-failure test. The M–technique yielded a smaller graft diameter (8.4 ± 0.5 mm) compared to the statistically equivalent diameters of the three continuous loop techniques (8.9 ± 0.6 mm of Arthrex-tech group, 9.1 ± 0.4 mm of Kessler-tech group and 9.2 ± 0.6 mm of Double Z-Tech group). The continuous loop grafts formed by the Double Z-Technique showed outstanding performance among the tested techniques in terms of ultimate failure load (982 ± 121 N) and cyclic elongation (3.7 ± 1.0 mm). There was no significant difference between the four groups in cyclic stiffness. Of the assessed techniques, the Arthrex technique resulted in the lowest ultimate elongation (2.0 ± 0.7 mm), followed by the Double Z-tech (4.5 ± 1.8 mm), the M-tech (5.2 ± 3.9 mm), and the Kessler-tech (5.3 ± 2.4 mm). The Arthrex-tech group (5.98 ± 0.38 min) displayed the shortest graft preparation time, followed by the M-Tech (7.94 ± 0.58 min), Kessler-tech (9.03 ± 0.39 min) and Double Z-Tech (13.29 ± 1.14 min). Double Z-Tech can improve the construct of continuous loop tendon graft with regard to mechanical performance.

## Introduction

The anterior cruciate ligament (ACL) has an important stabilization function for tibiofemoral stability and is one of the most frequently injured structures of the knee joint^1^.

Untreated ACL rupture can be associated with the occurrence of knee osteoarthritis and knee instability^2^. Therapeutic strategies of ACL ruptures have received extensive attention from scholars and with the development of sports medicine and arthroscopic techniques, arthroscopic anterior cruciate ligament reconstruction (ACLR) has become the most common surgical strategy for the treatment of ACL rupture^3^.

Currently, mainstream ACLR techniques utilize autogenous lower limb tendon grafts (i.e. hamstring tendons, quadriceps tendon) and bone patellar tendon bone (BPTB) grafts with tendon grafts being the most common^4,5^. Fixation with these techniques is usually achieved with suspensory or interference screw fixation with suspensory fixation being preferred by many surgeons^6^. Whereas conventional cortical fixation devices provide fixed length suspension, adjustable loop fixation^7^ introduced the possibility of individual graft length adjustment and maximization of graft-bone contact^8^. Depending on the surgeon’s preference, adjustable loop fixation devices can be used for femoral-only as well as for femoral and tibial graft fixation. In the latter case, the tendon explant is folded twice over the two adjustable loop fixation devices and the two tendon ends are connected to form a continuous double-loop tendon graft. This graft configuration is in contrast to the tendon-graft configuration used for more conventional fixation techniques such as interference screw tibial fixation where the two tendon ends are “baseball- stitched” and most commonly folded twice to create a tendon graft folded in M-configuration. Whereas adjustable loop fixation devices may provide sufficient fixation strength and rigidity the biomechanical behavior of continuous double-loop tendon grafts has not been reported.

In this biomechanical study, we compared a frequently applied hamstring graft preparation technique used for tibial adjustable length suspensory graft fixation^7^ (Arthrex-Tech: GraftLink^TM^, Arthrex, Naples, FL) to a conventional M-configuration technique (M-Tech) used in tibial interference screw fixation. Meanwhile, we also explored modified graft suturing techniques (Kessler-tech and Double Z-Tech) designed to improve the construct with regard to mechanical performance in isolated biomechanical testing. It was hypothesized that continuous loop tendon grafts were superior to the M-Tech graft in mechanical properties. The second hypothesis was that a graft using Kessler-tech or Double Z-Tech can improve the construct of continuous loop tendon graft with regard to mechanical performance.

## Materials and Methods

### Specimen preparation

This study was performed in accordance with the National Institutes of Health guidelines for the use of experimental animals, and all animal protocols were approved by the Institutional Animal Care and Use Committee of University of Zurich. The Ethics Committee of Canton of Zurich approved the study protocol, including any relevant details. Testing was performed using 16 bovine calf tendons purchased from a local slaughterhouse, with structural and biomechanical properties similar to those of human hamstringtendons^9^. Specimens were preserved at −20 °C and defrosted at room temperature for 1 hour before graft preparation. During graft preparation and biomechanical testing, phosphate buffered saline (PBS; pH 7.4) was used to keep the tendon hydrated per 10 minutes. Excess soft tissues were removed from the surfaces of tendons before graft preparation. The tendons were cut in half along their long axis for the 4-folded grafts to have a diameter of approximately 8 mm.

### Grouping

The 32 tendon specimens were randomly divided into four groups: 4-strand graft using baseball stitching at both tendon ends to form a double-folded tendon graft in M-configuration (M-tech), continuous loop graft using the GraftLink^TM^ graft preparation technique (Arthrex-tech), continuous loop graft using Kessler technique (Kessler-tech), and continuous loop graft using Double-Z technique group (Double Z-tech).

### M-Technique graft preparation

**Figure 1:**
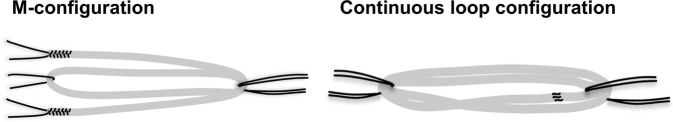
Schematic of the two tested tendon graft configurations.

As shown in Fig. 1, for the preparation of M-Tech grafts 20 mm of both tendon ends were whip stitched, the tendon was folded once over size 5 polyethylene suture (Meister & Cie AG, Bern, Switzerland), inserted into the femoral suspension loop, folded a second time to form a four-strand M-configuration tendon bundle and tied up using size 2 polyethylene suture (Orthocord, DePuy Mitek, Raynham, Massachusetts, USA).

### Continuous loop grafts

**Figure 2:**
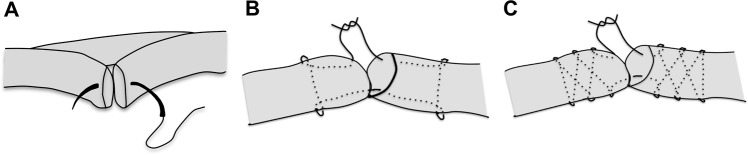
The three tested continuous loop configuration graft techniques. (**A**) Continuous loop graft using GraftLink^TM^ (Arthrex) suture technique; (**B**) continuous loop graft using Kessler anastomosis technique; (**C**) continuous loop graft using Double-Z anastomosis technique.

Continuous loop grafts were prepared by inserting the tendon into two looped size 5 polyethylene loading sutures (Meister & Cie AG, Bern, Switzerland) twice to form a double-loop continuous tendon graft (Fig. 1). The free tendon ends were then connected using one of the three tested suturing techniques (Arthrex-tech, Kessler-tech, Double Z-tech, Fig. 2). Once stitching was complete, the sutured tendon was moved to the inside of the construct and tensioned before applying the final circular contraction suture.

### GraftLink (Arthrex)technique

GraftLink (Arthrex)Technique was aided by use of guides for the continuous loop GraftLink (Arthrex, Naples, USA)^10^. Briefly, the graft was quadrupled symmetrically over two looped size 5 polyethylene loading sutures, and the free ends were whip-stitched and then sutured with a buried-knot technique^7^ (Fig. 2A).

### Kessler technique

To connect the free tendon ends the Kessler technique was applied^11^. The suture was hereby inserted longitudinally into the tendon core for a length of 10 mm. Then the suture was passed to the side of the tendon and reinserted into its core and brought back to the end of the tendon (Fig. 2B). After repeating these steps on the second free tendon end, the two suture ends were connected using a surgeon’s knot and three alternating overhand knots.

### Double-Z technique

**Figure 3:**
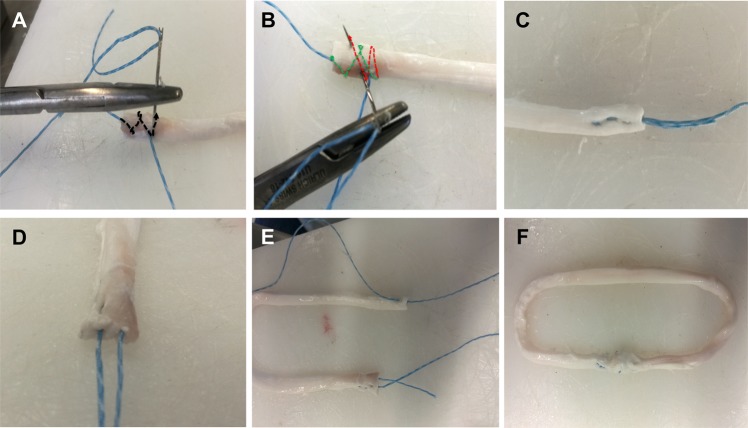
Continuous loop graft preparation technique using Double-Z anastomosis. (**A**) A needle with #2 non-resorbable surgical thread was inserted from one side of the tendon cross section and passed through the tendon four times achieving a Z shaped anastomosis; (**B**) the needle passed in the tendon reversely achieving the second Z shaped anastomosis. The green dotted line showed the first Z shaped anastomosis and the red dotted line showed the second Z shaped anastomosis; (**C**) elevation view of the suture; (**D**) cross-sectional view of the suture; (**E**) same method was performed to sew another free end; (**F**) a surgical knot was knotted between the both free ends achieving a continuous loop graft.

For Double-Z graft preparation two Z-shaped stitches were used on each of the two tendon ends as shown in Figs. 2C and 3. The four suture ends were then connected with a surgeon’s knot and three alternating overhand knots.

### Circular contraction suturing

**Figure 4:**
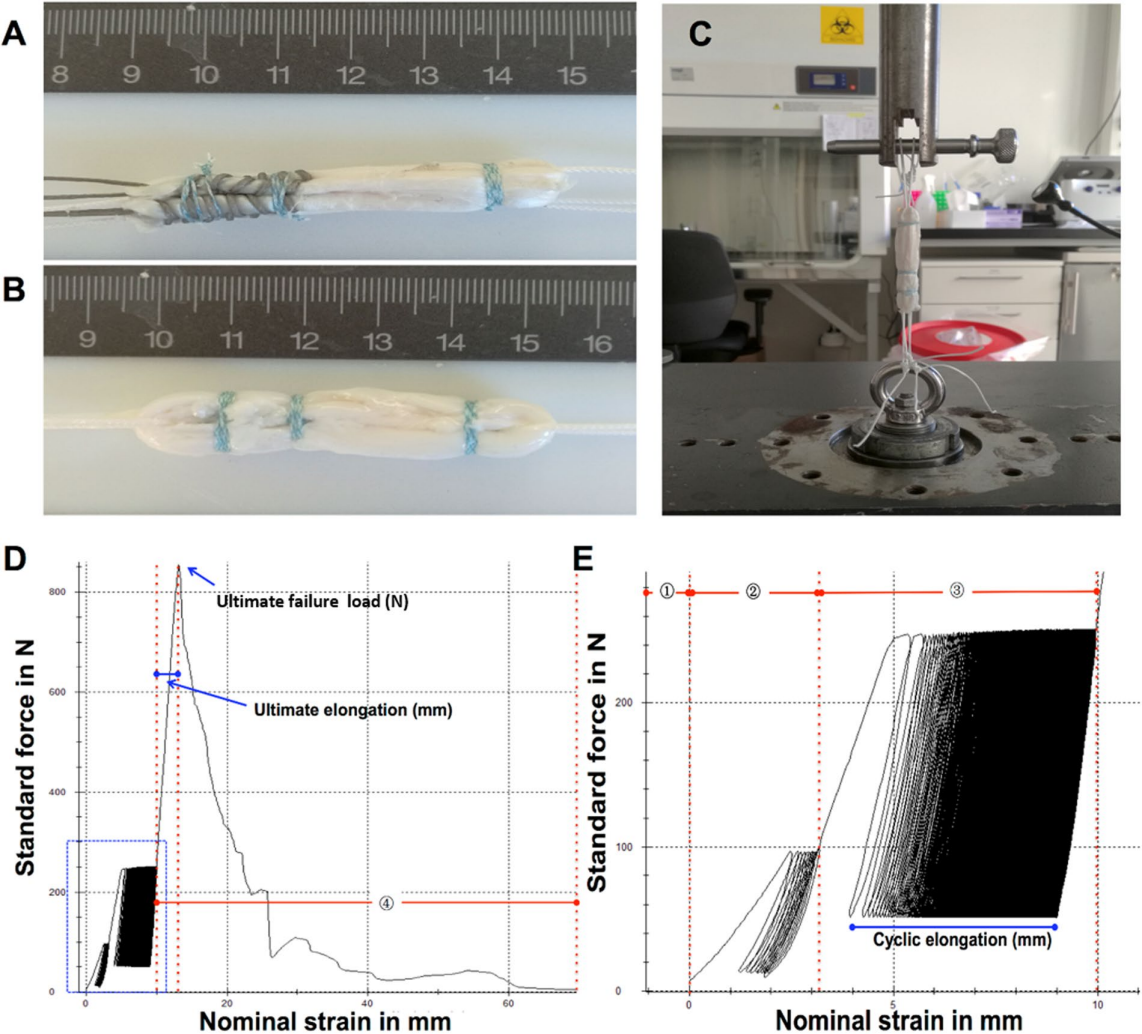
(**A**) M-configuration tendon graft; (**B**) Continuous loop tendon graft; (**C**) Biomechanical testing of a quadrupled continuous loop graft sample with a two double-looped loading ropes through the closed ends of the graft; (**D**) Typical test curves and parameterization. ④: ramp to failure phase; (**E**) enlarged view of blue area in Fig. 3D. ① Preload phase; ② preconditioning phase; and ③ cycling testing phase. Black arrows: Circular contraction sutures applied to increase tensile strength of the construct and reduce its diameter.

After connecting the free tendon ends using one of the above described techniques, the doubled continuous tendon loop was tied up using a slight modification of the technique described by Lubowitz *et al*.^7^ with the purpose of making the tendon graft more compact and increasing its stability. The connected tendon ends were hereby first moved to the inside of the graft. Size 2 non-resorbable suture was passed through the graft four times at a distance of 10 mm from the connection of the free tendon ends. First the suture was stitched from the inside of the graft to the surface, then it was brought through all tendon strands twice, wrapped around the graft twice and brought back from the outside in, back to the other suture end. The two suture ends were connected using surgeon’s knots and the buried knot technique^7^. This was repeated 10 mm from the connection of the free tendon ends in the opposite direction as well as on the opposing end of the tendon graft (Fig. 4A,B).

To determine the best location of three-contraction suture, a baseline biomechanical study was performed to analyze three different locations for the stitch end. GraftLink (Arthrex) technique was selected to connect the free tendon ends. One stitch location was near the loading suture at the outside the circular contraction sutures (group A); one stitch location was under that of the circular contraction suture (Group B) and in the middle of the two circular sutures (Group C). Each locations type contains 6 samples and was tested according to the following protocol.

### Biomechanical testing

Before mechanical testing, the diameter of the graft was measured using a graft sizing device (KARL STORZ SE & Co. KG, Tuttlingen, Germany). The tendon grafts were then placed into a universal testing machine (Zwick GmbH, Ennepetal, Germany) suspended with size 5 suture on both sides (Fig. 4C). After applying a preload of 10 N for 30 seconds and a subsequent preconditioning phase of 10 cycles from 10 to 100 N the graft was loaded between 50 and 250 N at a maximum rate of 1.5 mm/s for 5000 cycles^12,13^. The preconditioning protocol was aimed to simulate the loading regime the graft undergoes during surgical insertion. Then the tendon grafts were ramp loaded until failure at a machine speed of 20 mm/min^14^. Table 1 summarizes the specifics of the testing protocols.

**Table 1:** Mechanical testing definition.

Phase	Variable	Definition
Preload phase	Load	10 N
	Duration	30 s
	Positioning	Force controlled
Preconditioning phase	Mode	Cyclic loading
	Number of cycles	10
	Lower reversal point	10 N
	Upper reversal point	100 N
	Crosshead maximum velocity	1.5 mm/s
	Crosshead position over time	Linear
Test phase 1	Mode	Cyclic testing
	Number of cycles	5000
	Lower reversal point	50 N
	Upper reversal point	250 N
	Crosshead maximum velocity	1.5 mm/s
	Crosshead position over time	Linear
Test phase 2	Mode	Ramp to failure
	Failure definition	Force <0.7 × maximum force
	Crosshead maximum velocity	20 mm/min

### Biomechanical analysis

Force-displacement data was recorded using TestXpert 10 software (Zwick GmbH, Ennepetal, Germany) and processed in MATLAB (Matlab R2016a, Mathworks, Natrick, USA). Ultimate failure load (N) and ultimate elongation (mm) were defined as maximum force achieved and respective elongation with elongation zeroed at the end of preconditioning. Cyclic elongation (mm) describes total graft elongation during the 5000 test cycles. Cyclic stiffness (N/mm) was measured by averaging the slope of the linear curve connecting minimum and maximum force-position of each cycle (Fig. 4D,E).

### Failure mode

Sample testing was monitored by Dr. Chen Zhang and Tobias Götschi alternately and documented using a digital camera (EO Edmund, Tokyo, Japan). Four modes of sample failure were documented: Baseball stitch failure, loading ropes cut through tendon, continuous loop anastomosis (whipstitch, Kessler anastomosis and Double Z anastomosis) rupture, and continuous loop anastomosis loosening and subsequent structural failure during cyclic testing.

### Graft preparation time evaluation

Time required for the preparation of the graft using either of the techniques was recorded.

### Statistical analysis

Statistical results were generated by using a standard statistical software (GraphPad Prism6, CA, USA). Measurement data were expressed as the mean ± SD. Kolmogorov-Smirnov test was used to test if the values were adequately fit by Gaussian distributions. The measurement data were statistically analyzed using one-way analysis of variance for multiple group comparisons and the Dunnett t test for 2 group comparisons. The test level of *α* was 0.05.

### Ethics approval

The Ethics Committee of Canton of Zurich approved the study protocol.

## Results

### The best location for the stitch end

After the baseline biomechanical assessments, we found most of the stiches loosened and failed due to the pulling force of the loading loop when the graft securing sutures were located near the loading suture (6 of 6 in group A) and under the circular contraction sutures (5 of 6 in group B). No visible gap nor anastomosis loose was observed during cyclic testing in group C. Stitch location which was in the middle of the two circular sutures was thus chosen for the experiments in this study.

**Figure 5:**
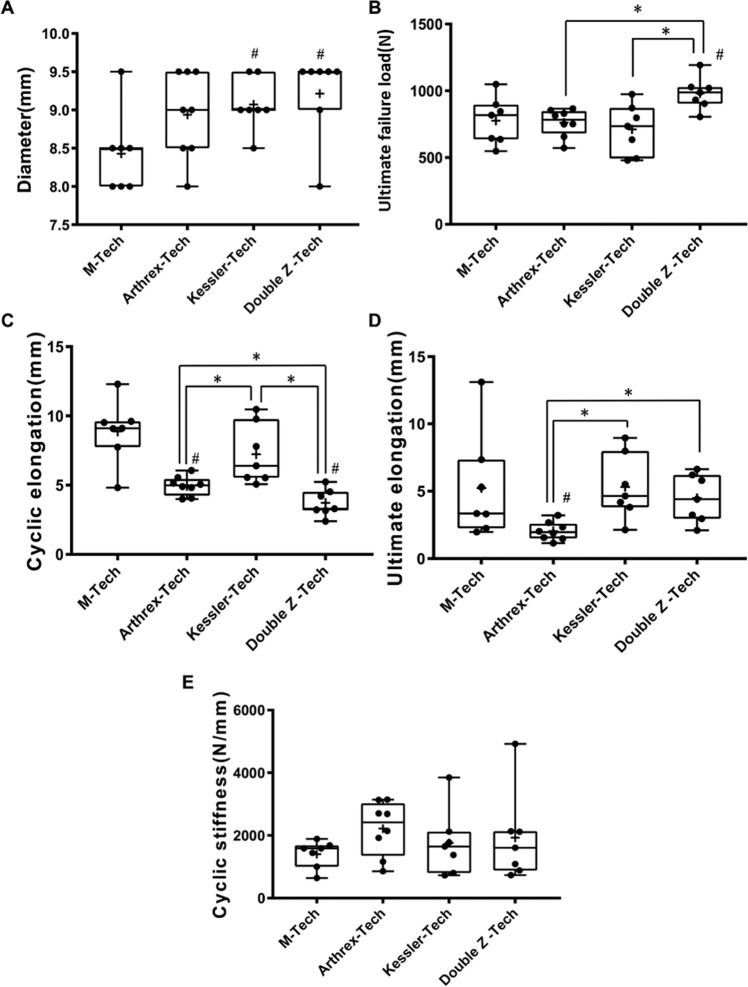
Box plots of mechanical test results. (^#^*P* < 0.05, compared with M-Tech group; ^*^P < 0.05, compared between 3 continuous loop grafts). (**A**) Diameter comparison; (**B**) ultimate failure load comparison; (**C**) cyclic elongation comparison; (**D**) ultimate elongation comparison; (**E**) cyclic stiffness comparison.

Each group of 8 graft specimens was mechanically tested and analyzed (Table 2, Fig. 5). The M-tech group and Double Z-tech group each lost one specimen due to data output failure from the test machine. In the Kessler-tech group, one graft experienced anastomosis loosening and failure during cycling testing rendering its mechanical parameters invalid for further analysis. Consequently, there were 29 specimens included in final analysis.

**Table 2:** Mechanical test results of different graft preparation suture technique.

Parameter	M-Tech (n = 7)	Arthrex-Tech (n = 8)	Kessler-Tech (n = 7)	Double Z –Tech (n = 7)
**Diameter, mm**				
Mean ± SD	8.43 ± 0.53	8.94 ± 0.56	9.07 ± 0.35	9.21 ± 0.57
Median	8.5	9	9	9.5
Min-Max	8–9.5	8–9.5	8.5–9.5	8–9.5
95% Confidence interval	7.93–8.92	8.47–9.41	8.75–9.39	8.69–9.74
**Ultimate failure load, N**				
Mean ± SD	777 ± 175.2	762.2 ± 103	712.3 ± 187.2	981.6 ± 121.1
Median	818.2	783.4	735.8	987.9
Min-Max	548.2–1049	572.3–866.9	479.3–973.9	805.7–1193
95% Confidence interval	615–939.1	676.1–848.4	539.2–885.4	869.6–1094
**Cyclic elongation, mm**				
Mean ± SD	8.88 ± 2.25	4.94 ± 0.69	7.23 ± 2.17	3.72 ± 0.97
Median	9.11	4.98	6.39	3.30
Min-Max	4.82–12.3	3.99–6.05	5.07–10.47	2.39–5.23
95% Confidence interval	6.80–10.96	4.36–5.53	5.22–9.24	2.82–4.62
**Ultimate elongation, mm**				
Mean ± SD	5.23 ± 3.94	2.04 ± 0.68	5.32 ± 2.40	4.48 ± 1.78
Median	3.35	1.95	4.65	4.41
Min-Max	1.97–13.10	1.15–3.21	2.14–8.95	2.10–6.63
95% Confidence interval	1.58–8.88	1.47–2.60	3.10–7.54	2.83–6.12
**Cyclic stiffness, N/mm**				
Mean ± SD	1405 ± 433.4	2219 ± 862.4	1758 ± 1051	1927 ± 1436
Median	1587	2416	1645	1609
Min-Max	640.7–1889	857.3–3142	729.2–3849	732–4921
95% Confidence interval	1004–1806	1498–2940	785.3–2730	598.5–3255

### Diameter

Each group of 8 graft specimens was mechanically tested and analyzed (Table 2, Fig. 5). The M-tech group and Double Z-tech group each lost one specimen due to data output failure from the test machine. In the Kessler-tech group, one graft experienced anastomosis loosening and failure during cycling testing rendering its mechanical parameters invalid for further analysis. Consequently, there were 29 specimens included in final analysis.

Analysis indicated that M-tech (M) group has the smaller diameter (8.43 ± 0.53 mm) than the three continuous loop graft groups (Fig. 5A). The mean diameter of Kessler-tech (K) group (9.07 ± 0.35 mm) and Double Z-tech (Z) group (9.21 ± 0.57 mm) were significantly greater than in the M-tech group (*t*_K/M_ = 2.673, *p* = 0.0203; *t*_Z/M_ = 2.668, *p* = 0.0205), whereas the diameter of Arthrex-tech (A) group (8.94 ± 0.56 mm) was not significantly different (*t*_A/M_ = 1.788, *p* = 0.0971). However, there was no significant difference among the three continuous loop graft groups (*t*_K/A_ = 0.5448, *p* = 0.5951; *t*_Z/A_ = 2.09, *p* = 0.0585; *t*_Z/K_ = 0.5695, *p* = 0.5795).

### Ultimate failure load

Optimized continuous loop grafts of Double Z –tech group (982 ± 121 N) had significantly higher ultimate failure load than conventional hamstring graft (777 ± 175 N) and continues loop graft using Arthrex-tech (762.2 ± 103 N) and Kessler-tech (712 ± 187 N) (*t*_Z/M_ = 2.542, *p* = 0.0259; *t*_Z/A_ = 3.794, *p* = 0.0022; *t*_Z/K_ = 3.196, *p* = 0.0077) (Fig. 5B). Failure strength of the other two continuous loop grafts was comparable to the conventional hamstring graft. No differences were found between Arthrex-tech group, the Kessler-tech group and the M -tech group (*t*_A/M_ = 0.2023, *p* = 0.8428; *t*_K/M_ = 0.6674, *p* = 0.5171; *t*_*K*/A_ = 0.6519, *p* = 0.5259).

### Cyclic elongation

The continuous loop grafts showed outstanding performance than hamstring graft in terms of cyclic elongation (Fig. 5C). The Double Z-tech group (3.72 ± 0.97 mm) displayed the smallest cyclic elongation compared to the M-tech group (8.88 ± 2.25 mm), Arthrex-tech group (4.94 ± 0.69 mm) and Kessler-tech group (7.23 ± 2.17 mm) (*t*_Z/M_ = 5.561, *p* = 0.0001; *t*_Z/A_ = 2.838, *p* = 0.014; *t*_Z/K_ = 3.899, *p* = 0.0021). The Arthrex-tech group also showed significantly better performance than M-tech group and Kessler-tech group (*t*_A/M_ = 4.711, *p* = 0.0004; *t*_A/K_ = 2.824, *p* = 0.0143). While cyclic elongation of the Kessler-tech group was the largest among the continuous loop groups, it was not statistically different to the conventional hamstring graft. No difference was observed between Kessler-tech group and M-tech group (*t*_K/M_ = 1.397, *p* = 0.1878).

### Ultimate elongation

The ultimate elongation of continuous loop grafts was not inferior to conventional hamstring graft preparation (Fig. 5D). The Arthrex-tech group (2.04 ± 0.68 mm) showed the smallest ultimate elongation compared to the M-tech group (5.23 ± 3.94 mm), the Kessler-tech group (5.32 ± 2.40 mm) and the Double Z-tech group (4.48 ± 1.78 mm) (*t*_A/M_ = 2.265, *p* = 0.0413; *t*_A/K_ = 3.721, *p* = 0.0026; *t*_A/Z_ = 3.612, *p* = 0.0032). No difference was found between Kessler-tech group, Double Z-tech group and M-tech group (*t*_K/M_ = 0.0503, *p* = 0.9607; *t*_Z/M_ = 0.4612, *p* = 0.6529; *tK*_/Z_ = 0.746, *p* = 0.47).

### Cyclic stiffness

Cyclic stiffness was highly variable in all groups, and no intergroup differences were observed (*F* = 1.416, p = 0.2428). The measured cyclic stiffness of Arthrex-tech group was 2219 ± 862 N/mm, the Kessler-tech group was 1758 ± 1051 N/mm, the Double Z-tech group was 1927 ± 1436 N/mm, and the M-tech group was 1405 ± 433 N/mm (Fig. 5E).

### Failure mode

Of the eight grafts that were tested in M-tech group, one was excluded due to data output failure, five failed in the form of baseball stitch failure and two of loading rope cut through. In the Arthrex-tech group, all of 8 samples failed by whipstitch rupture. Interestingly, 3 of 8 samples non-critically damaged with a visible gap forming at the 115^th^, 214^th^ and 2750^th^ cycle of cyclic testing. However, the graft structures were stable and survived to the end of the testing protocol. The Kessler-tech group showed the weaker anastomosis strength compared to the Arthrex group. Here, 4 of 8 samples similarly showed a visible gap within the free ends at early stages of cyclic loading (20^th^, 150^th^, 272^th^ and 963^th^ cycles), with one of the four grafts failing completely at the 2200^th^ cycle due to continuous loop anastomosis loose and structural failure. The remaining 7 samples completed the cyclic testing protocol failed as Kessler anastomosis rupture during ramp loading. Of the eight tested grafts in the Double Z-tech group, one was excluded due to data output failure, five failed in the form of Double Z anastomosis rupture and two from loading rope cut through. No visible gap nor anastomosis loose was observed during cyclic testing in Double Z-tech group.

### Graft preparation time

The Arthrex-tech group (5.98 ± 0.38 min) displayed the shortest graft preparation time compared to the M-tech group (7.94 ± 0.58 min), Kessler-tech group (9.03 ± 0.39 min) and Double Z-tech group (13.29 ± 1.14 min) (*t*_A/M_ = 7.952, *p* < 0.001; *t*_A/K_ = 15.52, *p* < 0.001; *t*_A/Z_ = 17.19, *P* < 0.001). While graft preparation time of the Double Z-tech group was the longest among the continuous loop groups, the mean graft preparation time of continuous loop groups (9.32 ± 0.68 min) was not statistically different to the M-tech graft (*t* = 1.153, *p* = 0.2581).

## Discussion

This study compared a frequently applied hamstring graft preparation technique used for tibial adjustable length suspensory graft fixation (Arthrex-tech) to a conventional M-configuration technique (M-tech) used in tibial interference screw fixation. Arthrex-tech grafts outperformed the more traditional M-tech in terms of cyclic- and ultimate elongation. In this work, we also explored modified graft suturing techniques designed to improve the construct with regard to mechanical performance in isolated biomechanical testing. Using a newly introduced Double-Z graft preparation technique (Double Z-tech) we could significantly increase ultimate failure load (by 28.9% compared to the Arthrex-tech) and reduce cyclic elongation of the continuous-loop construct (by 24.7% compared to the Arthrex-tech). To the authors’ knowledge, the current investigation is the first to functionally assess and compare these graft configuration techniques.

It was originally hypothesized that the diameters of continuous loop grafts and hamstring grafts would show no significant difference. However, the diameters of all three grafts were larger than hamstring graft, although there was no statistical difference between Arthrex-tech group and hamstring graft group. The reasons may be due to the continuous loop technology itself. As is known, the suture configuration ultimately determines the diameter of the entire graft. In fact, Arthrex-tech’s whip stitch suture is a side-by-side suture between the free ends of the tendon, which potentially increases the diameter of the suture configuration. However, due to the applied compression of the graft, the diameter of the tendon could be reduced by a circular contraction suture, leading to a lack of any significant differences in diameter between the Arthrex-tech group and the M-tech group. Both the Kessler-tech and the Double-tech Z group differ from the Arthrex-tech in that the suture passes through the interior of the tendon entirely, restricting compression of the tendon and consequently increasing the end diameter. Considerations for decreasing the diameter of the graft will be pursued in follow-up studies.

Elongation of the ACL graft should be kept to a minimum in order to avoid postoperative laxity. Our data indicated that continuous loop grafts showed better performance in terms of cyclic elongation and ultimate elongation compared to other grafts. In this respect, the Double Z-tech group and Arthrex-tech group outperformed the Kessler-tech group and M-tech groups. Notably, cyclic elongation in Double Z-tech group was superior to the Arthrex-tech group. This aspect may be useful in minimizing the widely-discussed windshield-wiper/bungee cord phenomenon and could help to potentially avoid postoperative laxity. Petre *et al*.^15^ reported a biomechanical result of cyclic elongation using the Arthrex technique with a tendon–button suspensory device named TightRope RT (Arthrex, Naples, USA). They reported a total elongation of 4.5 ± 0.7 mm after 1000 cycles (50 and 250 N). Our results using the Double Z-tech group and Arthrex-tech group are comparable - indicating that our implementation of the Arthrex technique can be considered as standard, and demonstrates that the Double Z-tech can provide superior technical performance in terms of ultimate failure load and cyclic elongation.

For the purpose of making the tendon graft more compact and increasing its stability, the doubled continuous tendon loop was secured using a three-suture technique. The actual location of three stitches were realized to be extremely important to the ultimate stabilization of the graft structure. We first performed a baseline biomechanical study analyzing three different locations for the stitch end. One stitch location was near the loading suture at the outside the circular contraction sutures; one stitch location was under that of the circular contraction suture and in the middle of the two circular sutures. In these biomechanical assessments we found most of the stiches loosened and failed due to the pulling force of the loading loop when the graft securing sutures were located near the loading suture and outside the circular contraction sutures. It should be noted that locating stitches beneath the circular contraction, while stabilizing the construct, can substantially enlarge the graft diameter. Locating the stitches in the middle of the two circular sutures yielded both good biomechanical performance and smaller diameters than other configurations, and was thus chosen for the experiments described in this study.

In this study, the Arthrex-tech group displayed the shortest graft preparation time while graft preparation time of the Double Z-tech group was the longest among the continuous loop groups. The reasons were due to the continuous loop technology and Double Z-tech themselves. As we introduced above, Arthrex-tech, M-tech and Kessler-tech were more mature and easy suture surgical techniques. In the sample preparation process, they were easier to implement. Because of the innovation and complexity of Double Z technology, it will take longer preparation time. However, the preparation time of these four surgical techniques were completed within 15 minutes, which was sufficient and reasonable for the preparation of the tendon for ACLR surgery.

During this time, the surgeon can complete surgical procedures such as joint cleaning, ligament exploration and bone bed preparation, without affecting the implementation of the operation. Therefore, although Double Z technology has the longest preparation time, it can still meet clinical needs. Considerations for decreasing the preparation time of the grafts will be pursued in follow-up studies.

Among the limitations to the present study, is the fact that bovine tendons rather than human hamstring tendons were used as a testing model. Bovine tendons yield a homogeneous graft size that allows the suturing method effects on ultimate implant size to be more accurately compared. Further, since no tendon rupture and no differences in stiffness were observed in the present study, it can be assumed that the differences between the groups were not affected by differences in tendon properties. Of course, such uniformly high-quality tendon does not reflect actual clinical practice. Third, as we discussed above, the diameter in continuous groups were unsatisfyingly large, and additional work will be required to refine the methods in a manner that minimizes increases in graft diameter. Finally, it is important to note that this *in vitro* study focuses only on mechanics of graft and neglects biological factors such as healing and incorporation of the graft into the surrounding bone tunnel. Additional intra-articular biomechanical experiments are planned to clarify some of these aspects.

Altogether, we hypothesized that continuous loop tendon grafts were superior to the M-Tech graft in mechanical properties and the Double Z-Tech can improve the construct of continuous loop tendon graft with regard to mechanical performance. Our data indicated that continuous loop grafts showed better performance in terms of cyclic elongation and ultimate elongation compared to other grafts. In this respect, the Double Z-tech group outperformed the Kessler-tech group and M-tech groups. Our findings confirm our hypothesis and oriented a new approach for continuous loop tendon-graft preparation. Future work is warranted to exploit potential performance increases that can be achieved by improved graft design.

## Conclusions

This study shows that continuous loop tendon grafts perform equivalently to the standard M-folding technique in cyclic and ramp-to-failure testing. We introduce a novel “Double Z” technique that biomechanically outperforms other preparation techniques, albeit with the drawbacks of being more time-consuming and yielding slightly larger overall graft diameters.

## Data Availability

The datasets generated during and/or analyzed during the current study are available from the corresponding author on reasonable request.
